# 1,5-Di­meth­oxy­naphthalene

**DOI:** 10.1107/S1600536813026731

**Published:** 2013-10-19

**Authors:** Emmanuel Marfo-Owusu, Amber L. Thompson

**Affiliations:** a Department of Chemistry, University of Cape Coast, Cape Coast, Ghana; b Chemical Crystallography, Department of Chemistry, Chemistry Research Laboratory, University of Oxford, Mansfield Road, Oxford OX1 3TA, England

## Abstract

The title compound, C_12_H_12_O_2_, lies across an inversion centre. The mol­ecular structure suggests that the meth­oxy groups in the 1- and 5-positions of the naphthalene moiety do not significantly distort the planar conformation of the ring system, which has a maximum deviation of 0.0025 (9) Å. In the crystal, mol­ecules pack in a herringbone arrangement in layers parallel to (100) and with chains propagating along [101] formed by very weak C—H⋯O inter­actions.

## Related literature

For details of the uses of 1,5-di­meth­oxy­naphthalene, see: Ashton *et al.* (1991 ▶); Amabilino & Veciana (2003 ▶); Kim *et al.* (2008 ▶); Kato *et al.* (2003 ▶); Rawson *et al.* (2006 ▶). For related compounds, see: Allen & Kirby (1984 ▶); Beintema (1965 ▶); Belskii *et al.* (1990 ▶); Bolte & Bauch (1998 ▶); Cosmo *et al.* (1990 ▶); Cruickshank (1957 ▶); Gaultier & Hauw (1967 ▶); Pawley & Yeats (1969 ▶); Rozycka-Sokolowska & Marciniak (2009 ▶); Rozycka-Sokolowska *et al.* (2004 ▶, 2005 ▶); Wiedenfeld *et al.* (1999 ▶); Wilson *et al.* (1996 ▶); Wilson (1997 ▶). For details of the low-temperature device used, see: Cosier & Glazer (1986 ▶). For details of the H-atom treatment, see: Cooper *et al.* (2010 ▶). For Cambridge Structural Database, see: Allen (2002 ▶).
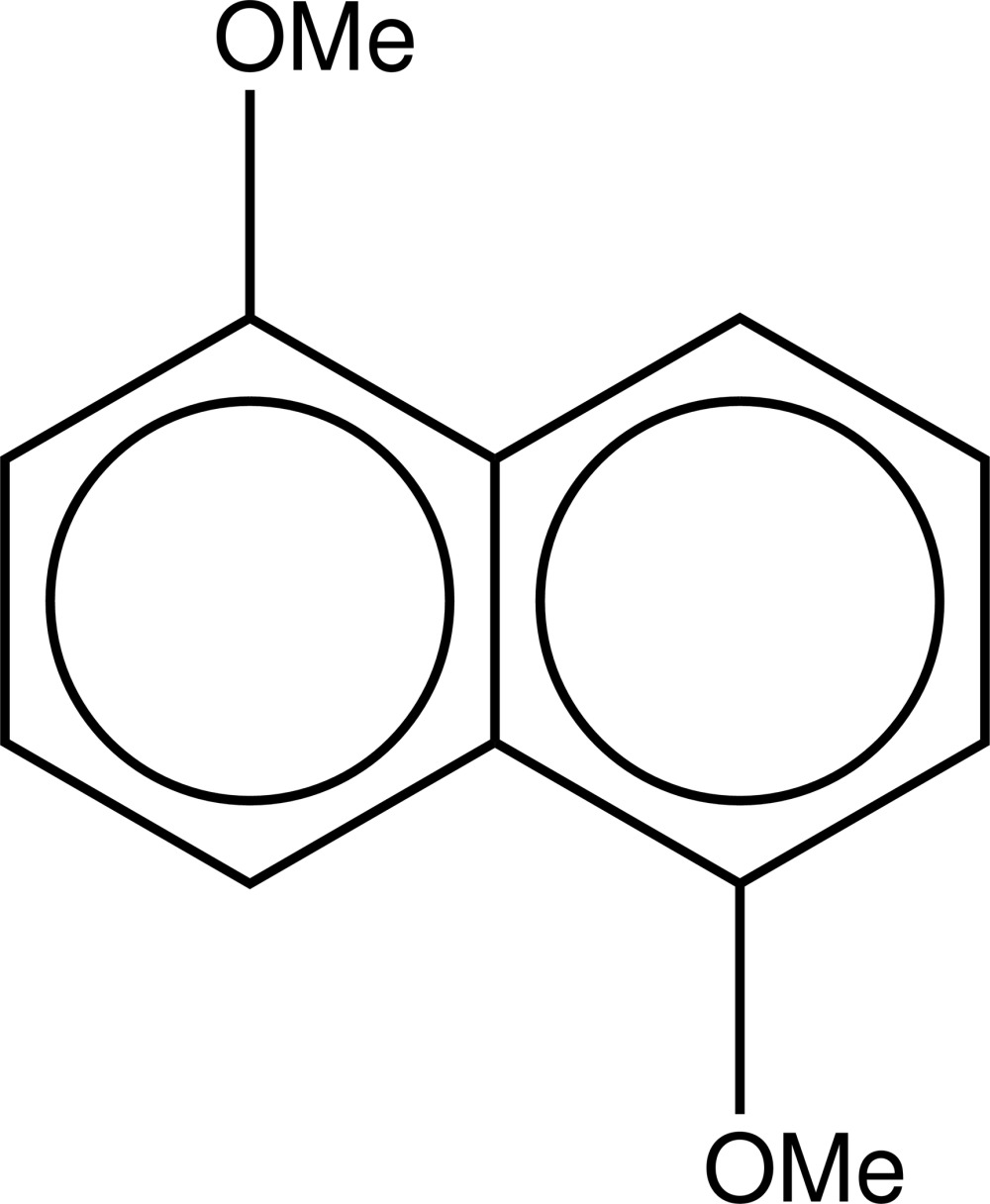


## Experimental

### 

#### Crystal data

C_12_H_12_O_2_
*M* *_r_* = 188.23
Monoclinic, 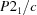

*a* = 7.0412 (3) Å
*b* = 10.1058 (4) Å
*c* = 6.5773 (2) Å
β = 95.509 (3)°
*V* = 465.86 (3) Å^3^
*Z* = 2
Cu *K*α radiation
μ = 0.73 mm^−1^
*T* = 150 K
0.18 × 0.08 × 0.01 mm

#### Data collection

Oxford Diffraction SuperNova diffractometer
Absorption correction: multi-scan (*CrysAlis PRO*; Oxford Diffraction, 2007 ▶) *T*_min_ = 0.59, *T*_max_ = 1.00
7624 measured reflections
975 independent reflections
890 reflections with *I* > 2σ(*I*)
*R*_int_ = 0.027

#### Refinement

*R*[*F* ^2^ > 2σ(*F* ^2^)] = 0.034
*wR*(*F* ^2^) = 0.093
*S* = 1.00
971 reflections
64 parameters
H-atom parameters constrained
Δρ_max_ = 0.22 e Å^−3^
Δρ_min_ = −0.18 e Å^−3^

### 

Data collection: *SUPERNOVA* (Oxford Diffraction, 2007 ▶); cell refinement: *CrysAlis PRO* (Oxford Diffraction, 2007 ▶); data reduction: *CrysAlis PRO*; program(s) used to solve structure: *SUPERFLIP* (Palatinus & Chapuis, 2007 ▶); program(s) used to refine structure: *CRYSTALS* (Betteridge *et al.*, 2003 ▶); molecular graphics: *CAMERON* (Watkin *et al.*, 1996 ▶); software used to prepare material for publication: *CRYSTALS*.

## Supplementary Material

Crystal structure: contains datablock(s) I, global. DOI: 10.1107/S1600536813026731/lh5655sup1.cif

Structure factors: contains datablock(s) I. DOI: 10.1107/S1600536813026731/lh5655Isup2.hkl

Supplementary material file. DOI: 10.1107/S1600536813026731/lh5655Isup3.cml

Additional supplementary materials:  crystallographic information; 3D view; checkCIF report

## Figures and Tables

**Table 1:** Hydrogen-bond geometry (Å, °)

*D*—H⋯*A*	*D*—H	H⋯*A*	*D*⋯*A*	*D*—H⋯*A*
C7—H73⋯O6^i^	0.98	2.70	3.495 (1)	139

Symmetry code: (i) 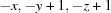.

**Fig. 1.**
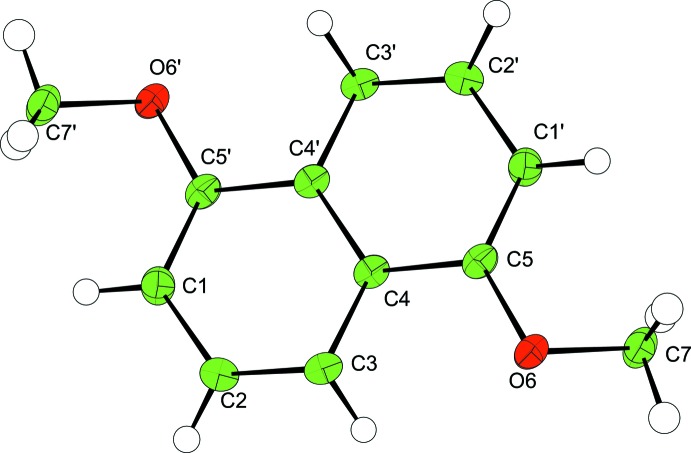
The title compound with displacement ellipsoids drawn at the 50% probability level. H atoms are shown as spheres of arbitary radius [symmetry operator indicated by a prime is -x + 1, -y + 1, -z + 2].

**Fig. 2.**
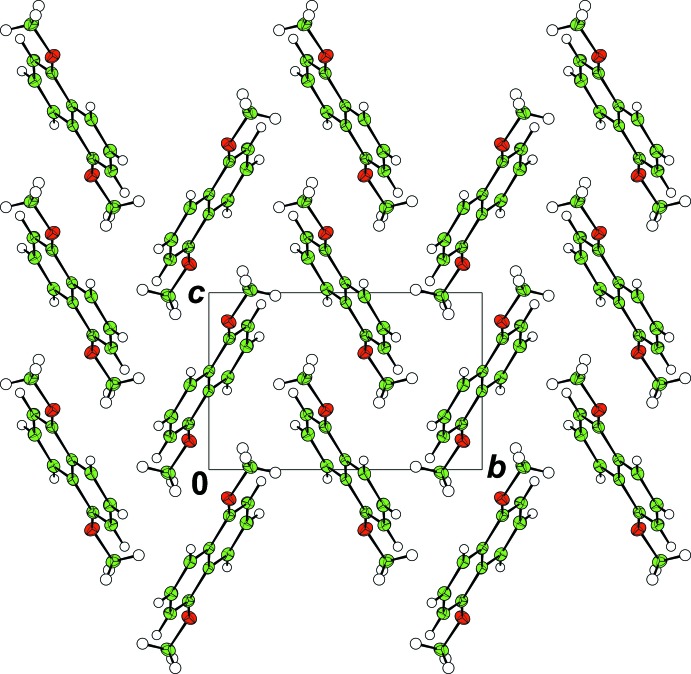
The packing in (I) viewed along [100] and showing the herringbone arrangement.

**Fig. 3.**
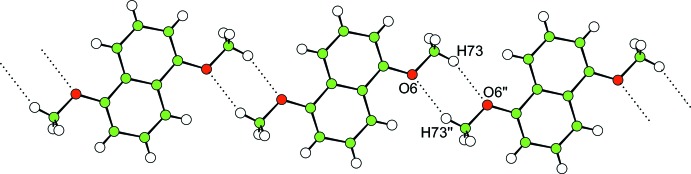
Intermolecular C—H···O hydrogen bonds forming chains that propagate along [101] (symmetry operator indicated by a double prime is -x, -y + 1, -z + 1).

## supplementary crystallographic information

### 1. Comment

1,5-Dimethoxynaphthalene has numerous industrial applications and uses. It is employed in the synthesis of pesticides (Kim *et al.*, 2008) for the agriculture industry, involved in the preparation of polyhydric alcohols (Kato *et al.*, 2003), as well as a component in molecular magnetic devices (Ashton *et al.*, 1991) for the electronics industry. It is also involved in the synthesis of more complex paramagnetic supramolecular architectures including rotaxanes and catenanes (Amabilino & Veciana, 2003, Rawson *et al.*, 2006). Despite this, reports discussing single-crystal studies of naphthalene (Pawley *et al.*, 1969; Wilson *et al.*, 1996; Wilson *et al.*, 1997) and its analogues including naphthol (Rozycka-Sokolowska, *et al.*, 2004; Rozycka-Sokolowska *et al.*, 2009; CSD (Allen, 2002) refcode NAPHOLO1), 1,4- and 1,5-dihydroxynaphthalene (Gaultier, *et al.*, 1967; CSD refcode NPHHQU10), Belskii *et al.*, 1990; CSD refcode VOGRUE) and 1,4-dimethoxynaphthalene (Wiedenfeld, *et al.*, 1999; CSD refcode ALUJIA; Cosmo *et al.*, 1990; CSD refcode MATFES) confirm that the crystal structure of 1,5-dimethoxynaphthalene (I) is not known.

The colorless single-crystal of 1,5-dimethoxynaphthalene was crystallized while attemping to crystallize the *rac*-1,1'-bi-2-naphthol/1,5-dimethoxynaphalene complex from a blend of methanol/ethylacetate solvents. It crystallizes in the monoclinic space group *P*2_1_/*c* with the molecule located on an inversion centre. The refined molecule and the labeling scheme are given in Fig. 1. It exhibits a herringbone packing motif and the molecules are arranged in layers parallel to the lattice plane (100) as shown in Fig. 2. All bond distances and angles fall within expected ranges.

In 1,5-dimethylnaphthalene (Gaultier, *et al.*, 1967; Belskii *et al.*, 1990; Beintema, 1965) as well as those in 1,4-dimethoxynaphthalene (Wiedenfeld, *et al.*, 1999), 1,8-dimethoxynaphthalene (Cosmo *et al.*, 1990), the steric interactions of the methyl groups cause a deviation from planarity in the naphthalene moiety. However, the ten-membered aromatic ring formed by atoms C1–C10 in (I) is planar; the steric interactions of the methoxy and H atoms do not cause any significant deviation from planarity. The exterior C4—C5—C4' angle (122.13 (9)°; symmetry operator indicated by a prime is -*x* + 1, -*y* + 1, -*z* + 2) in the naphathalene moiety shows no evidence of distortion in the naphthalene core associated with 1,5-disubstitutions. This suggests that the methoxy group seems to be restrained in the packing structure as a result of steric interaction between methoxy group and hydrogen atoms that reduce the propensity of the methoxy group to freely rotate in the crystal structure.

The methoxy substituents point away from the centre of the naphthalene moiety and each one forms a weak hydrogen bonded dimer with the neighbouring molecule. Since the molecule sits on an inversion centre, this leads to the formation of chains in the [101] direction (Fig. 3) *via* the weak intermolecular C—H···O hydrogen bonds involving the methoxy groups (with a C···O distance of 3.495 (1) Å).

In conclusion, the structure suggests that the methoxy groups in 1 and 5 positions around the naphthalene moiety do not significantly distort the planar conformation of the naphthalene, and the size of the groups and their positions are not influenced by steric interactions with the naphthalene moiety.

### 2. Experimental

The crystal of 1,5-dimethoxynaphthalene was obtained as a result of attemping to crystallize crystal complex of 1:1 mixture of *rac*-1,1'-bi-2-naphthol/1,5-dimethoxynaphalene from mixture of methanol and ethylacetate.

### 3. Refinement

The H atoms were all located in a difference map, but those attached to carbon atoms were repositioned geometrically. The H atoms were initially refined with soft restraints on the bond lengths and angles to regularize their geometry (C—H in the range 0.93–0.98, O—H = 0.82 Å) and *U*_iso_(H) (in the range 1.2–1.5 times *U*_eq_ of the parent atom), after which the positions were refined with riding constraints (Cooper *et al.*, 2010).

### Crystal data

**Table:**

C_12_H_12_O_2_	*F*(000) = 200
*M**_r_* = 188.23	*D*_x_ = 1.342 Mg m^−^^3^
Monoclinic, *P*2_1_/*c*	Cu *K*α radiation, λ = 1.54180 Å
Hall symbol: -P 2ybc	Cell parameters from 3622 reflections
*a* = 7.0412 (3) Å	θ = 4–77°
*b* = 10.1058 (4) Å	µ = 0.73 mm^−^^1^
*c* = 6.5773 (2) Å	*T* = 150 K
β = 95.509 (3)°	Plate, clear_pale_colourless
*V* = 465.86 (3) Å^3^	0.18 × 0.08 × 0.01 mm
*Z* = 2	

### Data collection

**Table:**

Oxford Diffraction SuperNova diffractometer	890 reflections with *I* > 2.0σ(*I*)
Graphite monochromator	*R*_int_ = 0.027
ω scans	θ_max_ = 76.7°, θ_min_ = 6.3°
Absorption correction: multi-scan (*CrysAlis PRO*; Oxford Diffraction, 2007)	*h* = −8→7
*T*_min_ = 0.59, *T*_max_ = 1.00	*k* = −12→12
7624 measured reflections	*l* = −8→8
975 independent reflections	

### Refinement

**Table:**

Refinement on *F*^2^	Primary atom site location: other
Least-squares matrix: full	Hydrogen site location: difference Fourier map
*R*[*F*^2^ > 2σ(*F*^2^)] = 0.034	H-atom parameters constrained
*wR*(*F*^2^) = 0.093	Method = Modified Sheldrick *w* = 1/[σ^2^(*F*^2^) + (0.06*P*)^2^ + 0.1*P*], where *P* = (max(*F*_o_^2^,0) + 2*F*_c_^2^)/3
*S* = 1.00	(Δ/σ)_max_ = 0.0004116
971 reflections	Δρ_max_ = 0.22 e Å^−^^3^
64 parameters	Δρ_min_ = −0.18 e Å^−^^3^
0 restraints	

### Fractional atomic coordinates and isotropic or equivalent isotropic displacement parameters (Å^2^)

**Table:**

	*x*	*y*	*z*	*U*_iso_*/*U*_eq_	
C1	0.46630 (14)	0.35777 (9)	1.31198 (15)	0.0246	
C2	0.28469 (14)	0.36191 (10)	1.19978 (15)	0.0251	
C3	0.25474 (13)	0.43124 (9)	1.02089 (14)	0.0226	
C4	0.40796 (12)	0.50104 (9)	0.94482 (13)	0.0202	
C5	0.38406 (14)	0.57492 (9)	0.75848 (14)	0.0219	
O6	0.20285 (10)	0.57222 (7)	0.66380 (11)	0.0274	
C7	0.16539 (15)	0.64994 (10)	0.48270 (16)	0.0296	
H11	0.4837	0.3088	1.4350	0.0297*	
H21	0.1819	0.3157	1.2519	0.0309*	
H31	0.1296	0.4338	0.9479	0.0276*	
H73	0.0325	0.6342	0.4324	0.0427*	
H72	0.1849	0.7431	0.5170	0.0424*	
H71	0.2476	0.6232	0.3793	0.0432*	

### Atomic displacement parameters (Å^2^)

**Table:**

	*U*^11^	*U*^22^	*U*^33^	*U*^12^	*U*^13^	*U*^23^
C1	0.0273 (5)	0.0242 (5)	0.0220 (4)	−0.0007 (4)	0.0001 (4)	0.0018 (3)
C2	0.0220 (5)	0.0258 (5)	0.0277 (5)	−0.0029 (4)	0.0041 (4)	0.0001 (4)
C3	0.0179 (5)	0.0236 (5)	0.0260 (5)	−0.0001 (3)	−0.0002 (3)	−0.0027 (3)
C4	0.0195 (5)	0.0186 (4)	0.0220 (4)	0.0012 (3)	−0.0002 (4)	−0.0031 (3)
C5	0.0210 (5)	0.0214 (5)	0.0225 (5)	0.0011 (3)	−0.0023 (4)	−0.0024 (3)
O6	0.0223 (4)	0.0314 (4)	0.0268 (4)	−0.0028 (3)	−0.0064 (3)	0.0060 (3)
C7	0.0298 (5)	0.0308 (5)	0.0261 (5)	−0.0002 (4)	−0.0077 (4)	0.0044 (4)

### Geometric parameters (Å, º)

**Table:**

C1—C5^i^	1.3717 (14)	C4—C4^i^	1.4236 (17)
C1—C2	1.4143 (13)	C4—C5	1.4312 (13)
C1—H11	0.946	C5—O6	1.3653 (11)
C2—C3	1.3683 (14)	O6—C7	1.4301 (11)
C2—H21	0.953	C7—H73	0.975
C3—C4	1.4197 (13)	C7—H72	0.974
C3—H31	0.962	C7—H71	0.972
			
C5^i^—C1—C2	119.63 (9)	C3—C4—C5	122.01 (9)
C5^i^—C1—H11	120.5	C4—C5—C1^i^	121.13 (9)
C2—C1—H11	119.9	C4—C5—O6	114.09 (8)
C1—C2—C3	121.39 (9)	C1^i^—C5—O6	124.78 (9)
C1—C2—H21	118.6	C5—O6—C7	117.29 (8)
C3—C2—H21	120.0	O6—C7—H73	106.7
C2—C3—C4	119.86 (9)	O6—C7—H72	109.1
C2—C3—H31	120.1	H73—C7—H72	110.2
C4—C3—H31	120.1	O6—C7—H71	110.8
C4^i^—C4—C3	119.90 (10)	H73—C7—H71	109.5
C4^i^—C4—C5	118.09 (11)	H72—C7—H71	110.5

Symmetry code: (i) −*x*+1, −*y*+1, −*z*+2.

### Hydrogen-bond geometry (Å, º)

**Table:**

*D*—H···*A*	*D*—H	H···*A*	*D*···*A*	*D*—H···*A*
C7—H73···O6^ii^	0.98	2.70	3.495 (1)	139

Symmetry code: (ii) −*x*, −*y*+1, −*z*+1.
